# The value of multicultural research: an interview with Mashaal Sohail on human genomics and creating representative genomic resources

**DOI:** 10.1038/s42003-022-03086-5

**Published:** 2022-02-18

## Abstract

Dr. Mashaal Sohail is an Associate Professor at the Center for Genome Sciences of the National Autonomous University of Mexico. Dr. Sohail received her PhD from Harvard University and completed a transnational post-doctoral research fellowship at the National Laboratory of Genomics for Biodiversity in Mexico and the University of Chicago. In this Q&A, Dr. Sohail tells us about her current work, experiences in building the Mexico BioBank, and the importance of diverse and transdisciplinary research.

**Figure:**
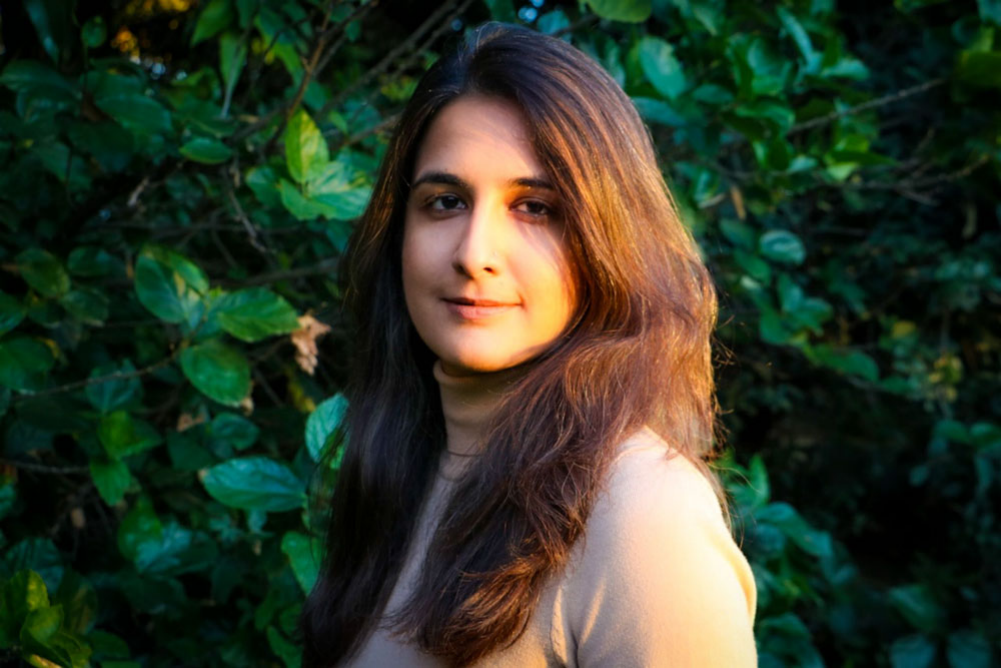
Andrew Philip Donaldson

Please tell us about your academic background and research interests.

I am a Pakistani scientist. I came to the US for my undergraduate studies at MIT. As required for all undergraduates, I had to take a biology course, which luckily for me, was taught by Dr. Eric Lander. That was my introduction to biology, and I ended up majoring in biological engineering. I was most excited by the computational work, though I also first learned to program at MIT. Introduced to evolution at the age of 21, I finally found something I was drawn to do research in. I began work in Dr. Jeff Gore’s lab to study the evolution of cooperation using inter-species experiments with yeast and bacteria. While enjoying the conceptual aspects, I was drawn more to quantitative work and had the honor of being accepted to do a PhD in the program in Systems Biology at Harvard. Allowed to join any lab at Harvard, after doing rotations that included analyzing cultural evolution using google ngrams and behavioral genetics experiments in worms, I wanted to analyze something concrete like genetic data using quantitative and computational tools. I decided on Dr. Shamil Sunyaev’s lab, where I got to train and work in distinct areas in population and human genetics, as well as have the liberty to pursue master’s studies in the history of science focusing on the history of evolutionary thought during my PhD. I was extremely lucky and privileged to be able to design a transnational post-doc which involved training in Dr. Andres Moreno-Estrada’s lab at Langebio, Cinvestav in Mexico and in Dr. John Novembre’s lab at the University of Chicago. My post-doc allowed me to venture deeper into understanding and helping solve the practical challenges inherent in genomics work with underserved groups and the conceptual challenges involved in doing complex trait genetics across diverse groups. I bring these distinct training experiences and insights to my own lab, where my group is focused on studying evolutionary processes and complex trait variation in humans and other species using computational genomics with implications for biomedicine. We retain an interest in related conceptual and practical issues of sustainable reciprocity with study subjects, and in historical relationships between genetics, race and racism and the role they play in our work today.

Your lab tackles many different topics, ranging from methods development to investigating the impact of medicinal plant usage. How do you balance these distinct research programs, and what is the benefit of this interdisciplinary work?

My interests have been, at least since the start of graduate school, in both evolution and neuroscience. I acquired training in developing and using methods and frameworks from population and statistical genetics to ask questions using genetic data about evolution and health. In the recent research projects I am planning, the goal is to continue similar genetics projects, as well as to explore newer projects using acquired skills from statistical, population and computational genomics to ask questions about the evolution and genetic architecture of traits including psychiatric traits. Important new themes are studying the effect of environmental perturbations on biology, and being inclusive in our research program of different people and of different medicinal frameworks.

In a framework where traits reside on a spectrum, I have tendencies that at an extreme can become attention deficit disorder. This kind of research program allows me to assuage those tendencies and to instead use them as a power that enables creation of distinct research programs within the same laboratory and of discovering new connections between them.

I believe it is exciting to consider what we may learn from opening more doors between fields and areas of study. Given the extreme specialization of the last century, I am of the school of thought that more dialogue and trans-disciplinary coursework and research will only be in the betterment of knowledge creation.

Why are initiatives like the Mexico Biobank important, and what tips would you give to investigators trying to build similar resources?

Initiatives like the Mexican Biobank are important for several reasons. First, the sustained development of scientific practice in the global south is a necessity right now, and this will help the continued development of genomic resources for everyone. Initiatives like the Mexico Biobank, which was firmly rooted and led in Mexico, help build local scientific capacity in the developing world, and increase representation in genomics through improved resources and training of local scientists. Second, more datasets capturing genetic and phenotypic diversity in different environments are necessary to make inferences about human genetic history, adaptations, and gene by environment interactions within the general research theme of the genetic architecture and evolution of complex traits and diseases across human diversity.

A team consisting of local as well as international players can be key. For the Mexican Biobank, a scientific team at the Mexican National Laboratory of Genomics for Biodiversity (of which I was part) partnered with the Mexican National Institute of Public Health and generated the genetic and phenotypic data locally, while creating collaborations with experts in the UK and US for developing shared collaborative expertise. Further tips to researchers building similar resources would be to be patient, be prepared for setbacks, and to learn to do more with less. It is a long road, and it is not easy to develop research infrastructure and projects from scratch, but it is extremely rewarding to indeed do so and to reap the fruits in research projects that will grow exponentially after a slow start and in the creation of a diverse scientific workforce that will only multiply. As in our case, it can also be promising to seek out existing national sampling initiatives and bring in a new genomics angle.

What do you think are the current impediments to research involving underrepresented populations in genetics?

One of the main impediments is that the scientific workforce and infrastructure is not well distributed across countries, ethnicities and economic status. As we build roads to assuage this situation, we can move towards sustainable research that involves underrepresented populations. This involves funding projects that recruit and train diverse scientists, making sampling choices to create representative genomic resources within equitable reciprocity frameworks, and doing conceptual work on how to design and perform genetic studies across diverse human groups and societies.

How do you maintain a diverse and inclusive environment in your own research lab?

I am in a constant process to try to identify and open padlocks in my mind that makes me resistant to diversity and inclusion. Sadly, because of the way human history has played out, there are plenty of explicit and implicit ways in which we are raised in societies and families that are not equal, diverse or inclusive. I believe that each of us has to do this work within ourselves first, and this will naturally help to create the same in the world, and in my case, in my lab. I also make these goals and philosophy visible in my lab’s presentations, website and Twitter, highlighting my commitment to recruit and train scientists from all backgrounds and histories, and especially those with identities that have struggled in the mainstream. In the lab, I consciously try to take small steps to encourage people to feel comfortable being who they are and in bringing aspects of their background to their work and the lab. It is in small acts of language we use, action and dress we permit, and activities we do professionally and socially. Finally, as part of the training in the lab, we learn to celebrate and understand the value that a multicultural and diverse environment brings in terms of intellectual growth and breadth.

In addition to your many other achievements, you obtained a master’s degree in the History of Science. How does this training help you in your current research, and why are these types of courses important?

Currently, the fields of population genetics and human genetics (among others) are reckoning with a history that is deeply intertwined with ideas around race and racism. Further, it has become imperative to consider ethics in our research, and the field is attempting to build frameworks to do inclusive research that is representative of human diversity, and to attempt to return fruits of research discoveries to all. This involves consideration of new sampling and analysis designs, creating collaborations with equitable reciprocity and playing a role in the communication of the research results to the study subjects and public at large. My training in the history of science, along with my training in population and human genetics, places me quite well to consider these different issues and help move the field forward by reckoning with its history, and forging new paths forward. I can also pass this training on to the next generation of scientists. This kind of training as part of a graduate education is crucially needed to allow larger numbers of geneticists to be informed and able to critically assess and shape the field’s present and future in light of its past.

*This interview was conducted by Associate Editor George Inglis*.

